# Sub-additive (antagonistic) interaction of lacosamide with lamotrigine and valproate in the maximal electroshock-induced seizure model in mice: an isobolographic analysis

**DOI:** 10.1007/s43440-020-00117-y

**Published:** 2020-06-07

**Authors:** Jarogniew J. Łuszczki, Maria Kondrat-Wróbel, Mirosław Zagaja, Sławomir Karwan, Hubert Bojar, Zbigniew Plewa, Magdalena Florek-Łuszczki

**Affiliations:** 1 Department of Pathophysiology, Medical University, Jaczewskiego 8b, 20-090 Lublin, Poland; 2 Isobolographic Analysis Laboratory, Institute of Rural Health, Lublin, Poland; 3 Regional Specialized Children’s Hospital, Olsztyn, Poland; 4 Department of Toxicology and Food Safety, Institute of Rural Health, Lublin, Poland; 5 Department of General, Oncological and Minimally Invasive Surgery, 1st Military Clinical Hospital, Lublin, Poland; 6 Department of Medical Anthropology, Institute of Rural Health, Lublin, Poland

**Keywords:** Antiepileptic drug, Drug interactions, Drug antagonism, Isobolographic analysis, Maximal electroshock

## Abstract

**Background:**

Launching polytherapy with two or three antiseizure drugs (ASDs) in patients with epilepsy is still problematic. The choice of ASDs to combine them together is usually based on clinicians’ experience and it requires knowledge about mechanisms of action of the studied ASDs and their drug–drug interactions, whose nature may be favorable, neutral or unfavorable. To characterize three-drug interaction among lacosamide (LCM), lamotrigine (LTG) and valproate (VPA), the type I isobolographic analysis was used. The antiseizure effects of three-drug combination were analyzed in a model of maximal electroshock-induced seizures (MES) in albino Swiss mice.

**Materials and methods:**

The seizure activity in mice was evoked by alternating current stimulation (25 mA, 500 V, 50 Hz, 0.2 s). Both, the type I isobolographic analysis and the test of parallelism of dose-response effects of the ASDs were used so as to properly classify interaction among three ASDs, administered in a fixed ratio combination of 1:1:1.

**Results:**

The three-drug mixture of LCM, LTG and VPA at the fixed ratio of 1:1:1 protected the experimental mice from MES-induced seizures; however, the reported interaction was sub-additive (antagonistic; *p* < 0.01) with isobolography.

**Conclusion:**

The antagonistic pharmacodynamic interaction among LCM, LTG and VPA in the MES test in mice cannot be transferred to clinical settings and this unfavorable combination should not be recommended for patients with epilepsy.

## Introduction

Polytherapy in epilepsy is usually prescribed for patients, whose seizure attacks are not adequately controlled with currently available antiseizure drugs (ASDs) [1, 2]. Nowadays, some ASD combinations are frequently prescribed by doctors than other combinations because of accumulating experimental and clinical evidence confirming their efficacy in epilepsy patients [3, 4]. For instance, the combination of lamotrigine (LTG) with valproate (VPA) is highly recommended for epilepsy patients [5, 6]. Evaluation of the efficacy of ASD combinations in clinical conditions is sometimes difficult because of ethical restrictions and limitations, including troubles with enrolment of the patients with the same types of seizures, similar history of the disease and almost identical response to the applied treatment. Additionally, replacement of one inactive drug with another that will be effective is not easy in epileptic patients [7–9]. On the other hand, some ASD combinations might occur antagonistic and should not be used clinically in patients in order not to expose them to ineffective treatment. For instance, the combination of LTG with carbamazepine (CBZ) is widely known to produce antagonistic effects in epilepsy patients [10, 11]. In clinical settings, the most frequent manifestation of antagonistic interactions between ASDs is lack of control on seizure attacks. If the patients have still seizures, physicians are obliged to replace one inactive drug used in polytherapy with another more efficacious ASD [3, 4]. The antagonistic interactions are not commonly recognized by physicians because the lack of seizure control, despite the polytherapeutic use of ASDs in combination, is usually considered as symptoms of refractoriness/resistance in epilepsy.

To help physicians in their choice of ASD combinations, preclinical studies on animals can test various ASD combinations providing evidence, which of the examined combinations are beneficial, neutral or unfavorable. From a preclinical point of view, ASDs in combination produce pharmacodynamic interactions, whose nature may be synergistic, additive, neutral or antagonistic [12–14]. At present, the isobolographic analysis of interaction is thought to be a gold standard in preclinical studies, when evaluating efficacy of ASDs or candidate drugs in animals [15]. Undoubtedly, researchers using this method can exactly classify interactions occurring among the tested drugs. From a clinical standpoint, the most beneficial ASD combinations are those offering synergy with respect to their anticonvulsant effects [16]. In contrast, the most unfavorable ASD combinations are those producing antagonistic interaction in terms of seizure suppression. At present, experimental evidence from preclinical studies can help clinicians in choosing the most appropriate ASD combinations because interactions observed in animals are similar to those observed in patients. There exists a close correlation between types of interactions observed in animals and humans [15, 17]. In other words, synergistic interactions between ASDs observed in clinical settings are also synergistic in preclinical studies on animals. For instance, it has been reported that the combination of LTG with VPA was synergistic in epileptic patients [4, 18] and in mice subjected to the maximal electroshock-induced seizure (MES) test [19]. Similarly, the combination of LTG with topiramate was synergistic in epileptic patients [20] and in the mouse MES model [19]. Also the combination of gabapentin with CBZ was synergistic in epilepsy patients [4, 18, 21] and in the animals challenged with the MES test [22]. Combinations of levetiracetam with CBZ, oxcarbazepine, LTG or VPA were synergistic in both clinical settings [3, 4, 18, 21] and in the mouse MES model [23]. The above-mentioned facts testify that there are some similarities between the types of interactions observed in animals and humans.

Despite immense progress in our knowledge about pathophysiological processes resulting in epilepsy attacks, we still cannot prevent seizure attacks or cure the disease. We are obliged to provide the epileptic patients with the best treatment options, based not only on one drug, but also on two or more ASDs in combination [17, 21]. Additionally, a close cooperation among clinicians and researchers should promote the synergistic and additive combinations of ASDs to translate them from preclinical studies to clinical settings. Of note, the synergistic interaction between ASDs in clinical practice can manifest in epileptic patients as a full seizure control, which is usually associated with reduction of ASD doses. Beneficial combination of ASDs always requires reduction of drug doses [1–4]. It is difficult, however, to clinically confirm that the chosen combination of two ASDs is favorable because some different criteria can be used to define clinical synergy in epileptology. Generally, the widely accepted criteria for synergy in clinical practice are: (a) the reduction in seizure frequency by more than 50% and/or (b) complete elimination of seizures with the state of seizure freedom during a defined period of time (usually, two years) [24]. Amelioration in seizure control in clinical trials is usually defined as a reduction in seizure frequency by 50%, especially in patients with refractory epilepsy [25]. Sometimes, the state of seizure freedom for two years is considered as an undeniable proof confirming indirectly that the combination of ASDs is efficacious in patients with refractory epilepsy [26–28]. Although the clinical criteria of synergistic interaction between ASDs may differ, but they are always defined precisely as a beneficial outcome, before the clinical studies start. On the other hand, synergistic interactions between ASDs that produce favorable clinical outcomes are usually published by physicians, as a result of their therapeutic success. In contrast, the antagonistic interactions occurring among ASDs and producing clinical failure are neglected by clinicians, who consider such situation as their defeats.

Accumulating evidence indicates that the combination of VPA with LTG is beneficial in both preclinical studies on animals and clinical studies in patients with refractory epilepsy [5, 6, 19]. Relatively recently, lacosamide (LCM)—a functionalized amino acid has been licensed as a novel third-generation ASD. Its unique molecular mechanisms of anticonvulsant action related with slow inactivation of sodium channels give physicians a novel efficacious drug that is used in patients with epilepsy [29, 30]. Considering the fact that the two-drug combination of LTG with VPA is experimentally and clinically favorable, we tried to ameliorate the anticonvulsant effects of this combination by adding another (third) ASD with novel mechanisms of action in a hope to synergistically interact in animals in terms of suppression of convulsions in animals and potentiate the effects exerted by the combination of LTG with VPA.

The aim of this study was to determine the interaction profile among three ASDs (LCM, LTG and VPA) in the mouse MES model using type I isobolographic analysis of interaction as described earlier [31, 32]. The MES model in mice reflects tonic-clonic and focal seizures in humans [33]. The selection of LCM, LTG and VPA in this study was based on several rational presumptions, including: (a) effectiveness of these ASDs in terms of suppression of both, MES-induced seizures in animals and tonic-clonic seizures in epilepsy patients; (b) diverse molecular mechanisms of action of these ASDs; (c) previous preclinical studies so as to compare the obtained results with those, previously published.

## Materials and methods

### Experimental animals and drug administration

All procedures involving animals comply with the ARRIVE guidelines [34] and were approved by the respective local ethics committee in Poland. In this study, 112 adult male albino Swiss outbred mice (weighing 20–26 g) were used. More specifically, 11 groups per 8 mice were studied in the MES test when determining the median effective doses (ED_50_ values ± SEM) for LCM, LTG and VPA administered separately, and 3 groups per 8 mice when evaluating the median effective dose (ED_50 exp_ value ± SEM) for the three-drug mixture. Both LCM (Vimpat^®^ UCB Pharma, Belgium) and LTG (Lamictal^®^ Glaxo Wellcome, UK) were suspended in an aqueous (1%) solution of Tween 80 (Sigma-Aldrich, Poznan, Poland). In contrast, VPA (sodium salt; Sigma-Aldrich, Poznan, Poland) was dissolved in sterile saline. All the ASDs were administered systemically (*ip*) in a volume of 5 ml/kg body weight. LCM and VPA were injected 30 min and LTG 60 min prior to the MES test, as recommended elsewhere [35–39].

### Maximal electroshock-induced seizure (MES) test

The tonic hind limb extension, as a result of seizure activity in mice, was evoked by an alternating current stimulation (50 Hz, 25 mA, 500 V, 0.2 s) using auricular electrodes. By plotting the logarithms of increasing doses of the ASDs (when administered separately) with their respective probits of the antiseizure effects in the MES test, it was possible to determine median effective doses (ED_50_ values ± SEM) of the ASDs that suppress tonic-clonic seizures in 50% of the mice, as described earlier [40]. Similarly, by plotting the logarithms of increasing doses of the three-drug mixture of LCM, LTG and VPA (in the fixed ratio combination of 1:1:1) with their respective probits of the antiseizure effects in the MES test, it was possible to calculate the experimental median effective dose (ED_50 exp_ value ± SEM) for the mixture of LCM, LTG and VPA against electrically evoked seizures in the MES test, as described earlier [31, 41].

### Type I isobolographic analysis and statistical analysis

The type of pharmacodynamic interaction for three-drug mixture administered *ip* in the fixed ratio combination of 1:1:1 was assessed isobolographically, as described earlier [31, 36, 38, 41–43]. Verification of parallelism of dose–response lines of the studied ASDs when administered alone allowed to calculate the additive median effective dose (ED_50 add_ value ± SEM) for the three-drug mixture. The experimentally derived ED_50 exp_ and the theoretically calculated ED_50 add_ values were statistically compared with the unpaired Student’s *t*-test, as reported earlier [42, 44, 45].

## Results

### Anticonvulsant effects of the studied ASDs in the experimental animals

**Fig. 1:**
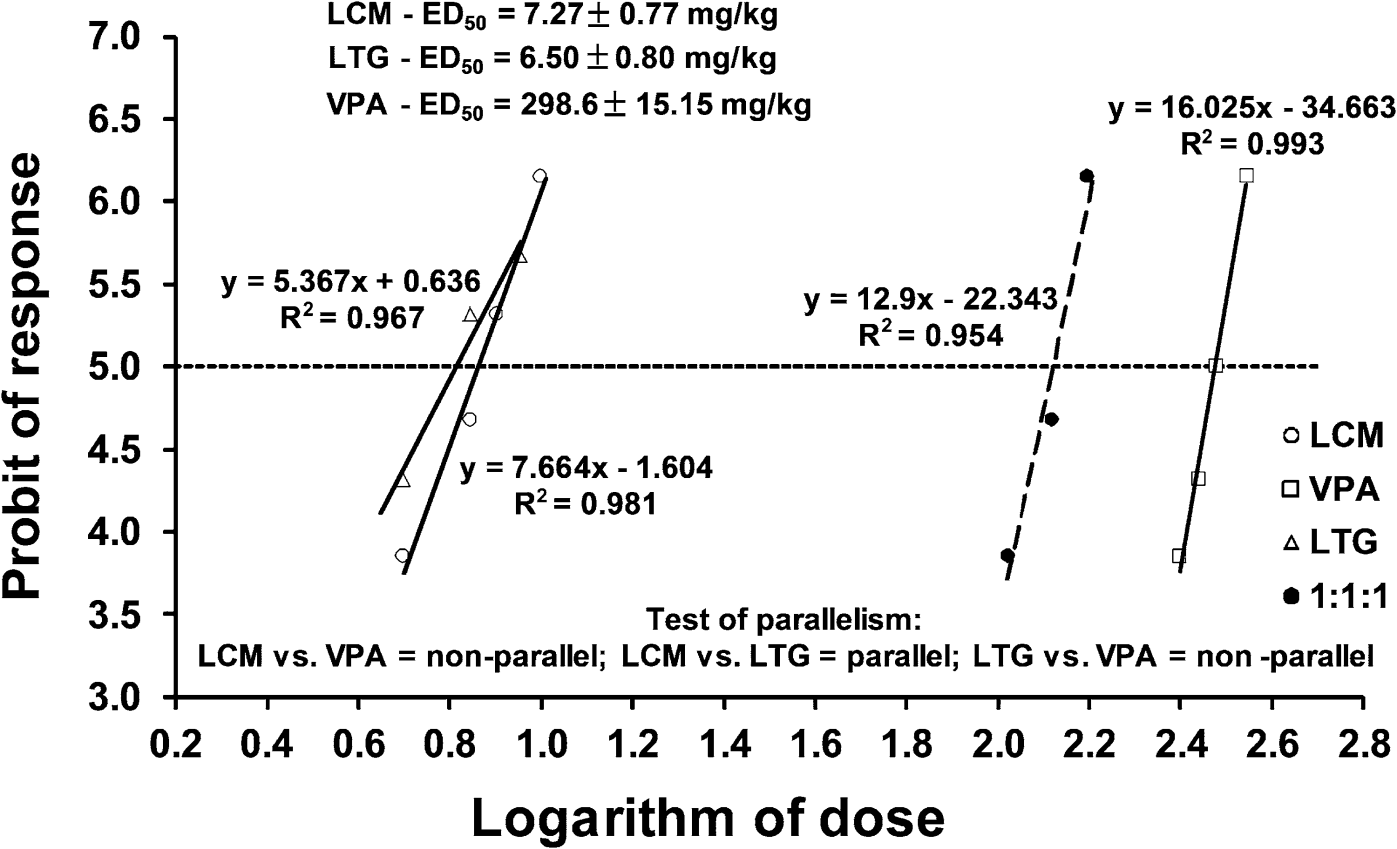
Dose–response effects of lacosamide (LCM), lamotrigine (LTG), valproate (VPA) and their combination (in the fixed ratio of 1:1:1) in the tonic–clonic seizure (MES) model in albino Swiss mice. Doses of LCM, LTG and VPA were transformed to logarithms (to the base 10) and plotted on *X* axis, while the anticonvulsant effects of the ASDs were transformed to probits and plotted on *Y*-axis of the Cartesian plot system. Dose–response effects of the ASDs were linearly related for LCM, LTG, VPA and their combination in the fixed ratio of 1:1:1. The ED_50_ values (± SEM) of LCM, LTG and VPA, along with the test of parallelism for the ASDs (according to log-probit method) are presented on the graph

The antiseizure effects of LCM, LTG and VPA in the MES test in mice allowed calculating their ED_50_ values (Fig. 1). The test of parallelism of dose–response effects proved that only LCM had its dose–response effect line collateral to that of LTG. On the contrary, VPA had its dose–response effect line non-parallel to that of LCM and LTG (Fig. 1). Lack of parallelism of dose–response effects for all the studied ASDs was responsible for testing only the mixture of three ASDs at the fixed ratio of 1:1:1, as recommended earlier [45, 46].

### Isobolographic analysis of interaction among three ASDs

With type I isobolographic analysis, a sub-additive (antagonistic) interaction was reported for the combination of LCM, LTG and VPA at the fixed ratio of 1:1:1 in the MES test in mice.

**Fig. 2:**
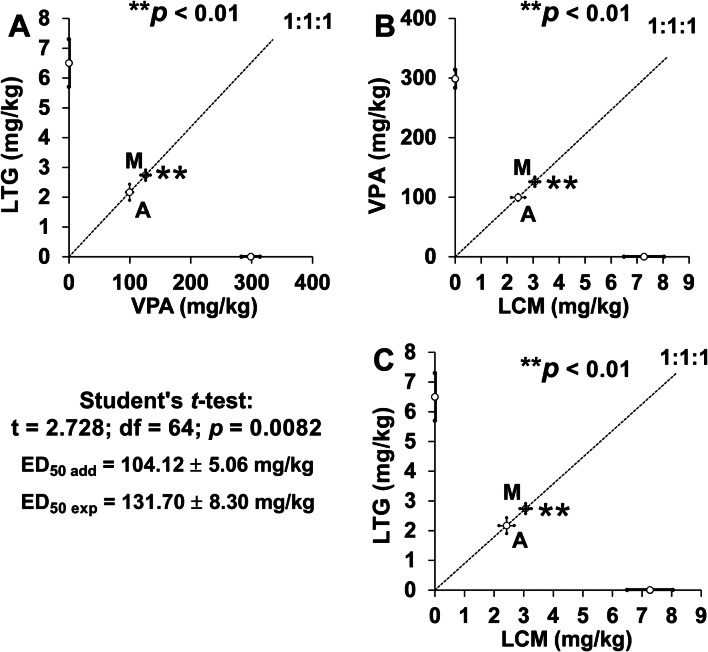
**a**–**c** Sub-additive (antagonistic) interaction among lacosamide (LCM), lamotrigine (LTG), and valproate (VPA), in the fixed ratio of 1:1:1 in the MES-induced seizure model in mice. Doses of ASDs are plotted on abscissa and ordinate of the Cartesian plot system, respectively. Points M and A on each graph illustrate the experimentally-derived ED_50 exp_ (± SEM) and the theoretically additive ED_50 add_ (± SEM) values, respectively. The point M is placed considerably above the point A (**p < 0.01), indicating sub-additive (antagonistic) interaction in the tonic–clonic seizure model in mice

The experimentally derived mixture that protected 50% of the animals tested (i.e., ED_50 exp_ = 131.70 ± 8.30 mg/kg) consisted of LCM in a dose of 3.07 mg/kg, LTG in a dose of 2.74 mg/kg and VPA in a dose of 125.89 mg/kg (Fig. 2a–c). The mixture of three ASDs that theoretically exerted additive protection of 50% of the mice tested (i.e., ED_50 add_ = 104.12 ± 5.06 mg/kg) comprised LCM in a dose of 2.42 mg/kg, LTG in a dose of 2.17 mg/kg and VPA in a dose of 99.53 mg/kg (Fig. 2a–c). The Student’s *t*-test revealed that both ED_50 exp_ and ED_50 add_ values significantly differed (*t* = 2.728; *df* = 64; *p* = 0.0082), indicating sub-additive (antagonistic) interaction in the mouse MES model (Fig. 2a–c).

## Discussion

Results presented in this study revealed that the tested three-drug combination of LCM, LTG and VPA exerted sub-additive (antagonistic) interaction in the mouse MES model and, thus, it produced effects that could not be as beneficial to patients as it would be theoretically expected. On the other hand, this study confirmed that only experimental evaluation gives us full reliable information about the exact types of interactions occurring among ASDs. From a rational viewpoint, physicians can combine ASDs with various molecular mechanisms of action to offer the patients better control of their seizures, especially for the patients with various seizure types and/or epileptic syndromes, which are refractory to the standard treatment [16]. On the contrary, ASDs with similar molecular mechanisms of action should not be combined together because of high risk of acute adverse effects that may occur during polytherapy [47]. In clinical practice, the ASDs are usually administered in full dose ranges, as in monotherapy [48, 49]. Thus, in the case of triple therapy, each patient takes three drugs in a fully active dose each. Very often, this “overtreatment” may evoke paradoxical seizures [50, 51]. However, to eliminate paradoxical seizures, the reduction of drug doses is necessary, but the main clinical problem is related with differentiation of paradoxical seizures from truly refractory epileptic seizures. At present, no uniform recommendation exists that could help clinicians face this problem.

In this study, it was found that the combination of LCM, VPA and LTG produced sub-additive (antagonistic) interaction in the mouse MES model. At present, there is no rational explanation trying to answer the basic question why LCM, VPA and LTG, when combined together, produce antagonistic interaction in the mouse MES model. On the other hand, there are some ASD combinations offering synergistic interaction with respect to suppression of tonic-clonic seizures in experimental animals (Table 1), and these combinations are highly recommended to be clinically used in patients with refractory epilepsy [4, 17, 21]. In the case of additive interactions among three ASDs (Table 1), these ASD combinations can also be efficacious in patients with epilepsy.

**Table 1:** Interactions for the studied three-drug combinations of antiepileptic drugs in the maximal electroshock-induced seizure test in mice

Combination of three antiepileptic drugs	Type of interaction	References
Lacosamide + lamotrigine + valproate	Infra-additive	(This study)
Lacosamide + carbamazepine + valproate	Infra-additive	[37]
Lacosamide + carbamazepine + lamotrigine	Additive	[36]
Lacosamide + carbamazepine + phenobarbital	Additive	[31]
Lacosamide + lamotrigine + phenobarbital	Additive	[35]
Carbamazepine + phenobarbital + valproate	Additive	[39]
Carbamazepine + phenobarbital + topiramate	Supra-additive	[41]
Oxcarbazepine + pregabalin + topiramate	Supra-additive	[38]
Phenobarbital + phenytoin + pregabalin	Supra-additive	[43]

A direct evaluation of interaction in clinical settings is, however, impossible because of huge numbers of ASD combinations theoretically available. Currently, physicians can use 25 various ASDs to treat epilepsy patients [8], and with these 25 drugs, one can expect 300 possible two-drug combinations. Simultaneously, the number of three-drug combinations (3 out of these 25 ASDs) increases up to 2300. So, it is unlikely to clinically verify the effectiveness of all the three-drug combinations. However, creation of an international data bank, where clinicians could deposit/find effective treatment regimens with three ASDs had been claimed several years ago [52]. It would allow selecting and choosing the recommended combinations. Such a data bank with recommended/suggested ASD combinations would help clinicians to choose the most effective treatment options for their patients. If a selected ASD therapy will be efficacious in one patient, information about such a combination will be available for other clinicians to treat similar seizures in other patients, even if there is no evidence-based medicine recommendation as yet [52, 53].

Results presented in this study indicated that by adding LCM to the synergistic combination of LTG with VPA in the mouse MES model, a decrease in the anticonvulsant effects of LTG and VPA was observed. Considering the three-drug interaction of LCM with LTG and VPA, one can expect synergy due to various molecular mechanisms of action of the studied ASDs. Instead of synergy, we observed antagonism and reduction of the anticonvulsant action of the combination in the mouse MES model. Of note, the two-drug combination of LTG with VPA produced synergistic interaction with respect to the protection from tonic-clonic seizures in both experimental mice and epileptic patients [5, 6, 18, 19].

Previously, an antagonism was also observed for the combination of LCM with CBZ and VPA in the mouse MES model [37]. In contrast, the combination of LCM with CBZ and LTG exerted additive interaction in the mouse MES model [36]. Another fact should be stressed while considering the rational selection of the ASDs for the three-drug combination in this study. It was observed that CBZ and VPA when combined together produced additive interaction in the mouse MES model [54]. In contrast, the two-drug combination of LTG with VPA in the mouse MES model was classified as synergistic [19]. Of note, CBZ and LTG although possess the similar mechanism of action related with fast inactivation of sodium channels in neurons, they also have additional anticonvulsant properties associated with the blockade of N-type and P/Q-type calcium channels by LTG [55], and the activation of adenosinergic system in the brain by interacting with adenosine A1 and A3 receptors by CBZ [56].

It is important to note that some three-drug combinations were verified clinically in patients with refractory epilepsy and in preclinical studies on animals [18, 41]. For instance, the combination of CBZ, phenobarbital and VPA was synergistic in both preclinical and clinical studies [18, 41]. Such comparison allowed us to confirm the existence of correlation of the effectiveness of the three-drug combinations between preclinical and clinical studies. These facts confirm and support the thesis that evaluation of types of interactions in preclinical conditions is justified. As already mentioned, translation of the results from preclinical studies to clinical settings needs a special attention related to doses of particular drugs used in mixtures. Of note, doses of the ASDs in this study corresponded to drug doses as determined in the MES test. In preclinical studies, doses of ASDs are reduced isobolographically to one-third of the effective doses of each drug used in the three-drug mixture at the fixed ratio of 1:1:1 [57]. Thus, the anticonvulsant effect produced by the mixture is always related to one ASD [44]. In contrast, in clinical practice during polytherapy, each drug is given to patients in the effective dose. Thus, patients on triple therapy usually take 3 drugs in full dose range each. This is the main difference between isobolographic studies and clinical settings.

The main limitation of this study is the acute (single) administration of the ASDs. Interactions observed isobolographically were evoked by drugs, which were administered acutely as *ip* single injections. No isobolographic interactions were determined after chronic administration of ASDs. Generally, during the chronic administration of the drugs, ASDs can exert some specific interactions associated with their influence on basic pharmacokinetic parameters related to absorption, distribution, metabolism and elimination of co-administered ASDs. Generally, the ASDs can mutually affect their metabolism by inhibiting and/or activating transformation of the ASDs into the inactive or active derivatives/metabolites. Additionally, some pharmacokinetic interactions can change final effects produced by drugs in the mixture. Thus, numbers of pharmacokinetic factors may affect the final effects produced by the ASDs administered chronically.

When combining three ASDs together, one can expect that some pharmacokinetic interactions among the tested drugs occur. However, in clinical trials, it has been reported that LCM did not affect pharmacokinetic content of VPA [58]. Similarly, LCM did not alter plasma levels of LTG in patients receiving both drugs [59]. Additionally, in the phase I of clinical studies, VPA had no impact on LCM plasma content [58]. Considering the above-mentioned facts, it is unlikely that LCM, VPA and LTG when combined together would be able to pharmacokinetically interact and mutually change their pharmacokinetic parameters. However, in this study, we did not measure total brain concentrations of ASDs because doses of three drugs used in the mixture from the MES test were low enough to be capable of significantly changing pharmacokinetics of LCM, LTG and VPA in experimental animals. Of note, doses of the ASDs (reflecting the ED_50 exp_ from the MES test at the fixed ratio combination of 1:1:1) were 3.07 mg/kg for LCM, 2.74 mg/kg for LTG, and 125.9 mg/kg for VPA, respectively. Similarly, acute adverse effects produced by the mixture were not determined in experimental animals in this study because of the low doses of the ASDs. Besides, it has previously been found that none of the tested three-drug combinations of ASDs in the mouse MES model displayed any signs of impairment of motor coordination, muscular strength or long-term memory in animals [31, 35–39, 41, 43]. Moreover, no acute adverse effects were observed for the combination of two-drug mixtures, including the mixtures of LTG with VPA [19], LCM with VPA and LCM with LTG [60]. Since the two-drug mixtures produced no side effects, the three-drug mixture of LCM, LTG and VPA would not be expected to produce side effects in the animals, especially, if doses of the ASDs were lower than those for the two-drug mixture.

The additional limitation of this study is the testing only one fixed ratio combination in the mouse MES model. Generally, the most favorable and preferentially tested fixed ratio combination is 1:1:1, i.e., when three drugs in mixture are used in the equi-effective doses, which exert the same quantitative effect [61], i.e., the anticonvulsant effect that protected the animals from MES-induced seizures. In this experimental seizure model, we determined the ED_50_ values protecting 50% of the animals tested against tonic-clonic seizures. Thus, the ED_50_ values of ASDs are considered to be the equi-effective doses, producing the same quantitative anticonvulsant effects in animals. The isobolographic analysis requires testing interaction among drugs that are used in doses exerting the same effects. Thus, each ASD in the mixture produces a comparable anticonvulsant effect in the seizure model [61].

In this study, the test of parallelism of dose–response relationship curves of the ASDs administered separately allowed us to conduct experiments only in one fixed ratio of 1:1:1. Lack of parallelism of dose–response relationship lines for the studied ASDs implies that only a fixed ratio of 1:1:1 can be tested isobolographically. Otherwise, the effects produced by the three-drug mixture at different fixed ratios than 1:1:1 could be over- or under-estimated [44, 45]. In such cases, the isobolographically-derived interactions would not be comparable. Besides, for the three-drug combinations different than 1:1:1, it is difficult to select one fixed ratio combination that could be tested preferentially because there are 6 different options of fixed ratios (including 1:3:1, 1:3:3, 3:1:1, 3:3:1; 3:1:3, or 1:1:3), where doses of particular drugs differ considerably. In such a case, experiments on animals require additional numbers of animals to be used. Unfortunately, according to the ARRIVE guidelines and the “3Rs” (Refinement, Reduction and Replacement) rule, related to testing on animals [34], we were obliged to keep the number of used animals as low as possible and the reduction of the number of animals tested in experimental conditions is obligatory. This was the main reason not to test other fixed ratio combinations in animals in this study.

## Conclusion

Summing up, the combination of LCM with LTG and VPA exerted antagonistic interaction in the mouse MES model. A special warning is required for patients treated with LTG, VPA and LCM because a sub-additive interaction would also be expected in patients receiving this ASD combination.
